# Dataset of Child schooling/ Out-of-school children in households in Kwara State, Nigeria

**DOI:** 10.1016/j.dib.2022.108654

**Published:** 2022-10-03

**Authors:** Adebayo Mohammed Ojuolape, Saidatulakmal Mohd

**Affiliations:** a Department of Economics, School of Social Sciences, Universiti Sains Malaysia, Malaysia; b Department of Economics, Faculty of Social Sciences, University of Ilorin, Nigeria

**Keywords:** Child schooling, Out-of-school children, Education enrollment, Education attainment

## Abstract

This article presents data from the survey of 1120 households across the three senatorial districts in Kwara State, Nigeria, in 2022. The data has information on the head of households, individuals in the households, household demographic characteristics, community characteristics, education, household head employment status, and the standard of living. The questionnaire includes both open-ended and close-ended questions.

The data is available in excel, and the coding explanation/ interpretation is presentable in a table.

This data is important for studies on child schooling enrollment, education attainment, and studies of United Nations Sustainable Development Goals, precisely goal four (UNSDG4).

**Specifications Table**

**Table:** 

Subject	Economics, Education, and Child Schooling
Specific subject area	Household child schooling, Out-of-school children in households, School enrollment and educational attainment in households.
Type of data	Table (The data is in excel format, also attached is a table explaining the data, and the questionnaire with the informed consent form)
How the data were acquired	This data was acquired with the use of questionnaires. This study adopted a random and systematic probability sampling technique. For the questionnaire, the households were randomly selected to give all households equal opportunity, as proposed by [1]. However, the communities were divided into areas for enumerations, with a specified number of questionnaires administered to each area. This is to ensure that there is no spatial dependence to ensure adequate representation. These two sampling techniques are considered because random sampling ensures representation, but it is not the best when considering spatial dependence, and on the other hand, systematic sampling is not representative, but it helps in reducing spatial dependence. Since systematic sampling is not representative but aids in minimizing spatial dependence while random sampling ensures representation but is not the best when considering spatial dependence, these two sampling techniques are used. Hence, using these two together ensures the study has a good sample (see; [2,3]). Questionnaire Design The objective of this study and theory served as a guide for the design of the research questionnaire and model specification. This aims to ensure that there is no omission of relevant information while analyzing data. The questions cover the head of households, individuals in the households, household demographic characteristics, community characteristics, education, employment status, the standard of living, and nature of dependents, among other things. The questionnaire includes both open-ended and close-ended questions. Measurement of Variables For Analysis, the variables were recoded to suit the specified models for the study's objective. Some of the categorical variables were changed to a binary form that is 0 and 1 as may be applicable, for example, gender, vocational training, among others, and ordered were coded as applicable. Dependent Variable Child Schooling was divided into enrolment and attainment. The determinants of enrolment show the variables that influence why some children have never enrolled, why some were enrolled and dropped out, and why others are currently enrolled. Also, attainment was used to show the factors determining the highest level of education.
Data format	Coded (The data has been coded and the modality for coding can be found in Table 1).
Description of data collection	This study used the 2006 Nigeria national population census, the most recent, to calculate the appropriate sample size. One thousand two hundred (1200) survey forms were prepared, and 400 were allocated for each local government. The head of household is the respondent for households. All the explanatory variables for the models specified were examined for inclusion in this study. The sample size is calculated with the formula below. $n=\;\frac{N}{1+N{\left(e\right)}^{2}}$ Where n is the sample size, N is the population size, and e is the level of precision. This is a simplified formula of [4] with the assumption that p, which is the proportion of variability, is (0.5), a confidence interval of 95%, and the margin of error is (0.05). The calculation showed 1189, which is sufficient sample size for this study as the required for the analysis of probability models, which are non-linear, is 500 samples size [5,6].
Data source location	• City/Town/Region: Ilorin, Offa, and Lafiagi. • Local Governments: Ilorin East, Ilorin West, Ilorin South, Offa, and Edu. • State: Kwara State. • Country: Nigeria.
Data accessibility	Repository name: Mendeley Data Data identification number (DOI):10.17632/82zygyrsk3.4. Direct URL to data: https://data.mendeley.com/datasets/82zygyrsk3/4

**Value of the Data**

• This data is useful to researchers in economics, education and related fields interested in analyzing how factors like household income and community factors affect child schooling enrollment or attainment.
• The data is useful for the government as this shows the situation of education/ schooling in households.
• This data can assist the government in planning and development. It can serve as a guide for implementing an education development plan and assessing the performance of existing universal basic education programs.
• This data will assist the United Nations in assessing the progress of the United Nations Sustainable Development Goals and also guide in knowing areas that need further attention.

## 1 Data Description

The data presented was collected between November 2021 and April 2022. The questionnaire was designed following the research objectives and theories while also building on the existing National bureau of statistics standard. This data can be accessed on mendeley repository [7]

The respondents are heads of households. Household members aged between 0 and 17 are regarded as children in households.

The coded data is presented in excel, and the explanation is also available in Table 1. The data includes 1120 households and 1972 children.

**Table 1:** Description of variables for Child schooling enrolment and child schooling attainment.

Variable	Definition	Measurement	Description
CSE	Child schooling enrolment	This is a polytomous response with three outcomes. 1 if never enrolled, 2 if was enrolled, and 3 is enrolled	This is the enrolment of school-aged children (0–17 years)
AGE	Age	Measured in years	This is a continuous variable from age 0–17 years
GEN	Gender	1 for males and 0 for female	This is the gender/sex of the child
PEA	Parental Education Attainment	1 for a minimum of secondary school education and 0 if otherwise	This is the highest level of education for the parents of school-aged children
PEMP	Parental employment status	This is a polytomous response, it is 1 if one parent is employed, 2 if both are employed and 3 if both unemployed	This is the employment status of the parents of the school-aged children
EDUCOST	Cost of Education	This is a continuous variable	The is the monetary cost of enrolling a child in school
HINC	Household Income	This is a continuous variable	This is the total monetary income of households
INCEN	Incentive for education	1 if there is a reward for enrollment and 0 if otherwise	This is either a monetary or other kinds of reward for schooling.
SIZE	Household size	The number of people in a household	This represents the number of people that live in a particular household.
HOHS	Head of Household Sex	Dummy variable with 1 for male and 0 for female	This shows whether the head of household under the survey is male or female
POLYGAMY	Polygamous Family	1 for polygamy and o otherwise	This shows whether the father of the school-aged children has just one wife or multiple wives.
TRIBE	Household tribe	A dummy variable with 1 for Yoruba and 0 if otherwise	This is to show if the household belongs to the majority tribe, which is Yoruba or otherwise.
BORDER	Birth order	This 1 for first child, 2 for middle children and 3 for last child	This shows the order of birth of the children in a household.
DISTSCH	Distance to school	This is a continuous variable	This shows the kilometers a child must cover to get to a school.
ACCESS	Access to school	1 if easily accessible and 0 if otherwise	This is to ascertain the level of difficulty in attending a school for school-aged children in the household.
RURAL	Rural Area	Dummy with 1 if urban and 0 for a rural location	This shows the level of development of the location of the household.
EDUDEC	Education Decision	1 for willing enrollment and 0 for compulsion	This is to ascertain the willingness of a school-aged child to acquire an education.
PEER	Peer Influence	1 if friends are enrolled and 0 if otherwise	This helps to show how the enrolment of peers in the environment encourages schooling of the other children
CSA	Child schooling attainment	This is a polytomous choice/response variable with five outcomes. 1 for uneducated, 2 for primary education, 3 for secondary education, 4 for graduate education and 5 for postgraduate.	This shows the highest level of education attained/completed by each child

Source: Author's formulation in line with the research question and theoretical and empirical literature.

## 2 Derivation and Sources of Variables

This study is inspired by the work of [8], where individual characteristics of the child such as age, gender, household income, family structure, birth order, and parental employment were emphasized, just like school availability and environmental factors were identified. The importance of age, number of children, parental education attainment and income, were stressed by [9], [10], [11], [12].

[13,14] elaborate on child characteristics, parental education, household characteristics, quality and cost of education, and gender, in determining child schooling. [15,16] also showed that individual household and community characteristics like education of the head of household, age, birth order, and family size influence schooling.

Older studies like [17] led to the inclusion of location as the study shows that location, sex, parental education, family structure, having a sibling, household income, and government policies on education determines child schooling, just like the study of [18].

Theories like rational action theory explain how gender, education decisions, and performance, among others, determine child schooling [19,20].

The human capital investment theory and the relative risk aversion under the rational action theory presented in [20,21] show how household characteristics affect human capital development, just like economic characteristics.

Variables that fall under environmental characteristics are linked to the two theories. Distance to school and the impact on the school were explored in [18].

### 2.1 Brief Statistical Overview

The average age of school-aged children is 11.9 years, the average household income is NGN 120,704.70 K, and the average annual cost of education per child is NGN 53,912.64. The average distance from school is 4.97 km. The data shows that there are more male children, and the tribe of most households surveyed are not Yoruba.

The data show that children that do not have access to secondary education are below 30%. The data also revealed that a higher number of children have dropped out of school as against the percentage enrolled, while about 11.26% never enrolled.

## 3 Experimental Design, Materials and Methods

The authors carried out a pilot study before and after approval of the ethics committee to ensure that all errors were corrected and to prevent potential misinterpretation in the questionnaire. The questionnaire contains questions that focus on the head of households, individuals in the households, household demographic characteristics, community characteristics, education, employment status, the standard of living, and nature of dependents, among other things.

### 3.1 Measurement of Variables

For Analysis, the variables were recoded to suit the specified models for each study objective. Some of the categorical variables were changed to a binary form that is 0 and 1 as may be applicable, for example, gender, vocational training, among others, and ordered were coded as applicable.

## Ethics Statements

Ethical clearance was approved for Household child schooling/ Out-of-school children by Universiti Sains Malaysia (USM) Research Ethics Committee (Human) (USM/JEPeM/21020151).

## CRediT authorship contribution statement

**Adebayo Mohammed Ojuolape:** Conceptualization, Methodology, Validation, Investigation, Resources, Data curation, Writing – original draft, Writing – review & editing. **Saidatulakmal Mohd:** Validation, Investigation, Writing – review & editing, Supervision.

## Declaration of Competing Interest

The authors declare that they have no known competing financial interests or personal relationships that could have appeared to influence the work reported in this paper. This research did not receive any specific grant from the public, commercial, or not-for-profit funding agencies.

## Data Availability

Dataset for Child schooling/ Out of school children in Kwara state, Nigeria (Original data) (Mendeley Data).

## Appendix Supplementary materials

Image, application 1
