# Supplementary material for: Dataset of Child schooling/ Out-of-school children in households in Kwara State, Nigeria
Source: Data Brief. 2022 Oct 3;45:108654. doi: 10.1016/j.dib.2022.108654 (PMC9679475; doi:10.1016/j.dib.2022.108654)
Supplement: Supplementary file 1 [file mmc1.docx]

**Questionnaire**

Dear respondent,

I seek your indulgence to kindly participate in filling/answering this questionnaire. I am a PhD student of Economics, School of Social Sciences, Universiti Sains Malaysia. I am conducting a household survey on the Household quality of life for my PhD thesis. The main purpose of this study is to analyse the determinants of Child schooling, Youth and Middle-aged unemployment, Elderly poverty and their overall effects on the quality of life in the households in Kwara State, Nigeria.

Kindly assist me by responding to few questions concerning you and your household members. If you agree to participate in this exercise, please endeavour to respond to all questions honestly.

The questionnaire would only take 25-30 minutes of your time. All your responses will remain safe and confidential. This is a voluntary exercise and you are free to decide to continue or withdraw at any time. You also do not have to respond to any question without your volition. Your cooperation is highly appreciated because without the support of people like you, it will be difficult if not impossible to carry out research like this.

Sincerely,

OJUOLAPE, Adebayo Mohammed

PhD Student,

School of Social Sciences,

Universiti Sains Malaysia,

Malaysia.

All households are to be represented by the head of households.

**Section A: Location**

1. Identification of Area; Local government-

Town-

1. Distance from Economic activities-
2. Address-
3. Length of stay-(Years)

Section B: Household Demographic characteristics

| 1 | 2 | 3 | 4 | 5 | 6 | 7 |
| --- | --- | --- | --- | --- | --- | --- |
|  | **Sex** | **Age** | **Relationship to head of household** | **Individual marital status** | **Individual religion** | **Individual tribe** |
|  | Male-------1 |  | Head-------------------------------1 | Married (monogamy)------1 | Muslim-------------1 | Yoruba------------1 |
|  | Female----2 |  | Spouse-----------------------------2 | Married (Polygamy)-------2 | Christian-----------2 | Fulani/Hausa----2 |
|  |  |  | Child-------------------------------3 | Divorced--------------------3 | Traditional---------3 | Nupe--------------3 |
|  |  |  | Grandchild------------------------4 | Separated--------------------4 | Others (Specify)---4 | Others------------4 |
|  |  |  | Siblings----------------------------5 | Widow(er)------------------5 |  |  |
|  |  |  | Nephew/Niece--------------------6 | Never married--------------6 |  |  |
|  |  |  | In-law------------------------------7 |  |  |  |
|  |  |  | Parent------------------------------8 |  |  |  |
|  |  |  | Parent In-law----------------------9 |  |  |  |
|  |  |  | Cousins---------------------------10 |  |  |  |
|  |  |  | Other extended relative---------11 |  |  |  |
| **People in household** |  |  | Servant----------------------------12 |  |  |  |
| **(Identification)** |  |  | Squatter---------------------------13 |  |  |  |
| 1------ |  |  |  |  |  |  |
| 2------ |  |  |  |  |  |  |
| 3------ |  |  |  |  |  |  |
| 4------ |  |  |  |  |  |  |
| 5------ |  |  |  |  |  |  |
| 6------ |  |  |  |  |  |  |
| 7------ |  |  |  |  |  |  |
| 8------ |  |  |  |  |  |  |
| 9------ |  |  |  |  |  |  |
| 10------ |  |  |  |  |  |  |

Section B: Household Demographic characteristics

|  | 8 | 9 | 10 | 11 | 12 | 13 |
| --- | --- | --- | --- | --- | --- | --- |
|  | **Individual current education status** | **Highest education attained** | **Vocational training** | **Religious education** | **Occupation/wages** | **Average household income (monthly)** |
|  | Never attended school--------------1 | None-----------------------1 | Yes------1 | Yes------1 | Fully employed in paid work with government--------------------------------1 |  |
|  | Attended but discontinued---------2 | Primary education-------2 | No------2 | No------2 | Fully employed in paid work with private organisation----------------------2 |  |
|  | Attending Primary School------3 | Secondary education----3 |  |  | Self-employed (Skilled)-----------------3 |  |
|  | Attending Secondary education---4 | Diploma-------------------4 |  |  | Self-employed (unskilled)--------------4 |  |
|  | Attending Tertiary education -----5 | Degree---------------------5 |  |  | Unemployed------------------------------5 |  |
| **People in household** |  | Postgraduate-------------6 |  |  |  |  |
| **(Identification)** |  |  |  |  |  |  |
| 1------ |  |  |  |  |  |  |
| 2------ |  |  |  |  |  |  |
| 3------ |  |  |  |  |  |  |
| 4------ |  |  |  |  |  |  |
| 5------ |  |  |  |  |  |  |
| 6------ |  |  |  |  |  |  |
| 7------ |  |  |  |  |  |  |
| 8------ |  |  |  |  |  |  |
| 9------ |  |  |  |  |  |  |
| 10------ |  |  |  |  |  |  |

**Section C: Child schooling specific**

| 1 | 2 | | 3 | 4 | 5 | 6 | 7 | 8 |
| --- | --- | --- | --- | --- | --- | --- | --- | --- |
|  | **Interest in education decision** | | **What is the educational performance** | **What is the highest level of the child's parents/guardian/Head of household education?** | **Individual child's parental/Head of household employment?** | **What is the average annual cost of education per child** | **Is there incentive for schooling?** | **Is there access to government universal basic education scheme?** |
|  | Does the (Individual) child show interest in acquiring education and going to school? | Are you willing and capable to sponsor your child/children to any level of interest? | How will you rate your child’s performance? | None-------------------1 | Fully employed in paid work with government---------1 |  | Yes-----1 | Yes-----1 |
|  | Yes------1 | Yes-----1 | Excellent ----------1 | Primary  education-------------2 | Fully employed in paid work with private organisation------------------------2 |  | No------2 | No------2 |
|  | No-----2 | No------2 | Very good---------2 | Secondary  education-------------3 | Self-employed (Skilled) ------------3 |  |  |  |
|  |  |  | Good---------------3 | Diploma--------------4 | Self-employed (unskilled)----------4 |  |  |  |
|  |  |  | Fair-----------------4 | Degree----------------5 | Unemployed--------5 |  |  |  |
| **People in household** |  |  | Poor----------------5 | Postgraduate--------6 |  |  |  |  |
| **(Identification)** |  |  |  |  |  |  |  |  |
| 1------ |  |  |  |  |  |  |  |  |
| 2------ |  |  |  |  |  |  |  |  |
| 3------ |  |  |  |  |  |  |  |  |
| 4------ |  |  |  |  |  |  |  |  |
| 5------ |  |  |  |  |  |  |  |  |
| 6------ |  |  |  |  |  |  |  |  |
| 7------ |  |  |  |  |  |  |  |  |
| 8------ |  |  |  |  |  |  |  |  |
| 9------ |  |  |  |  |  |  |  |  |
| 10------ |  |  |  |  |  |  |  |  |

**Section C: Child schooling specific**

|  | 9 | 10 | 11 | 12 | 13 | 14 | 15 | 16 |
| --- | --- | --- | --- | --- | --- | --- | --- | --- |
|  | **What is the birth order of the child?** | **Residential distance from the closest school** | **Is there access to secondary education in the area** | **Is there access to tertiary education?** | **Who are your child's friends? Are they enrolled?** | **Who are the parent's associates? Are their wards in school?** | **what is the marital status of child's parents?** | **Gender of child** |
|  |  |  | Yes-----1 | Yes-----1 | Yes-----1 | Yes-----1 | Married (monogamy)----1 | Male--------1 |
|  |  |  | No------2 | No------2 | No------2 | No------2 | Married (Polygamy)------2 | Female----2 |
|  |  |  |  |  |  |  | Divorced--------------------3 |  |
|  |  |  |  |  |  |  | Separated------------------4 |  |
|  |  |  |  |  |  |  | Widow(er)-----------------5 |  |
| **People in household** |  |  |  |  |  |  | Never married-------------6 |  |
| **(Identification)** |  |  |  |  |  |  |  |  |
| 1------ |  |  |  |  |  |  |  |  |
| 2------ |  |  |  |  |  |  |  |  |
| 3------ |  |  |  |  |  |  |  |  |
| 4------ |  |  |  |  |  |  |  |  |
| 5------ |  |  |  |  |  |  |  |  |
| 6------ |  |  |  |  |  |  |  |  |
| 7------ |  |  |  |  |  |  |  |  |
| 8------ |  |  |  |  |  |  |  |  |
| 9------ |  |  |  |  |  |  |  |  |
| 10------ |  |  |  |  |  |  |  |  |
